# Total disc replacement versus fusion for lumbar degenerative diseases - a meta-analysis of randomized controlled trials

**DOI:** 10.1097/MD.0000000000016460

**Published:** 2019-07-19

**Authors:** Deng-yan Bai, Long Liang, Bing-bing Zhang, Tao zhu, Hai-jun Zhang, Zhi-guo Yuan, Yan-fei Chen

**Affiliations:** a Department of Orthopedics, Second Provincial People's Hospital of GanSu, Lanzhou, Gansu Province; b Department of Orthopedics, Wangjing Hospital of China Academy of Chinese Medical Sciences, Beijing, China.

**Keywords:** lumbar degenerative diseases, lumbar fusion, meta-analysis, total disc replacement

## Abstract

**Background:**

Lumbar fusion is considered to the gold standard for treatment of spinal degenerative diseases but results in adjacent segment degeneration and acquired spinal instability. Total disc replacement is a relatively new alternative avoiding the occurrence of the above complications. The systematic review and meta-analysis was designed to evaluate whether total disc replacement exhibited better outcomes and safety.

**Methods:**

PubMed, Web of Science, Embase, the Cochrane Library, Chinese National Knowledge Infrastructure Database(CNKI), Wangfang database, and VIP database were searched for RCTs comparing total disc replacement with lumbar fusion. All statistical analyses were carried out using the RevMan5.3 and STATA12.0 software.

**Results:**

Of 1116 citations identified by our search strategy, 14 RCTs met the inclusion criteria. Compared to lumbar fusion, total disc replacement significantly improved ODI, VAS, SF-36, patient satisfaction, overall success, reoperation rate, ODI successful, reduced operation time, shortened duration of hospitalization, decreased postsurgical complications. However, total disc replacement did not show a significant difference regarding blood loss, consumption of analgesics, neurologic success and device success with lumbar fusion. And charges were significantly lower for total disc replacement compared with lumbar fusion in the 1-level patient group, while charges were similar in the 2-level group.

**Conclusion:**

Total disc replacement is recommended to alleviate the pain of degenerative lumbar diseases, improve the state of lumbar function and the quality of life of patients, provide a high level of security, have better health economics benefits for 1-level patients.

## Abstract 2

Supplemental Digital Content is available in the text

## 1 Introduction

Low back pain is a very common symptom. Globally, years lived with disability caused by low back pain increased by 54% between 1990 and 2015, with the biggest increase seen in low-income and middle-income countries. Low back pain is now the leading cause of disability worldwide.^[1,2]^ Lumbar degenerative disc disease is the major cause of low back pain.^[3,4,5]^ Lumbar degenerative disc disease is associated with genetic and environmental factors and affects many people around the world.^[6]^ With progressive degeneration, the effectiveness of the nutrition mechanism of the intervertebral disc decreases, in consequence, nucleus pulposus cells lose the ability to produce extracellular matrix proteins and proteoglycan, which results in disc progressive instability and desiccation.^[7]^

In patients suffering from chronic low back pain caused by lumbar degenerative disc disease, previous studies have shown that surgical treatment is more effective than conservative treatment in relieving low back pain.^[8]^ And fusion is the primary surgical option to treat disabling mechanical low back pain.^[9]^ Although fusion surgery yields better results in decreasing pain and disability compared to the conservative treatment, it also has detrimental effects on the normal physiological and biomechanical function of the spine.^[10]^

Total disc replacement is another surgical option approved in the mid-2000's for the treatment of lumbar disc herniation.^[11]^ The mechanism of pain relief is based on the combination of complete discectomy, restoration of segmental load transfer and sagittal balance and motion.^[12,13]^ Besides, a secondary intention of this technique is the preservation of normal motion at the adjacent lumbar levels, hoping that this will reduce later degeneration of the adjacent lumbar segments.^[14]^ Many clinical trials and follow-up studies of the use of lumbar total disc replacement have shown that total disc replacement is not inferior when compared with the standard spinal fusion procedures.^[15–17]^ Even more, renewed interest in disc arthroplasty has occurred in USA over the past decades and several groups have published encouraging results.^.^^[20,21]^ and total disc replacement was more common in younger patients.^[17]^ In addition, several previous meta-analyses have reported on this topic have different opinions on total disc replacement and lumbar fusion for treating lumbar degenerative diseases.^[46,48,49]^

Therefore, it was still uncertain whether total disc replacement was more effective and safer than fusion. The objective of this study was to systematically compare the efficacy and safety of total disc replacement to fusion for the treatment of lumbar degenerative disc diseases.

## 2 Materials and methods

The review protocol was registered with the International Prospective Register of Systematic Reviews (PROSPERO registration No.CRD42018112661), available online: http://www.crd.york.ac.uk/PROSPERO/display_record.php?ID=CRD42018112661). This article was written using PRISMA reporting guidelines and was based on previously conducted studies. Thus no ethical approval and patient consent are required.

### 2.1 Search strategy

As with the original review, we used the search strategies recommended by the Cochrane Back Review Group for the identification of RCTs.^[18]^ The literature were retrieved using multiple online databases including PubMed, Web of Science, Embase, the Cochrane Library, Chinese National Knowledge Infrastructure Database (CNKI), Wangfang database, and VIP database, for all years up to October 2018. There were no limits on study dates or any languages, publication types, and status restrictions. The key terms used in these searches were “total disc replacement”, “intervertebral disc replacement”, “artificial disc replacement”, “fusion”, “lumbar degenerative diseases”, “lumbar degeneration”, “spondylolisthesis”, “ lumbar disc herniation”, “lumbar disc protrusion”, “ lumbar spinal stenosis”, “ligamentum flavum hypertrophy”, “ligamenta flava thickening”. Different search strategies were used for Chinese and foreign language databases. In addition, the reference lists of previously published systematic reviews on the subject of total disc replacement versus fusion for lumbar degenerative diseases were manually examined for pertinent studies.

### 2.2 Inclusion criteria

The retrieved literature was screened by 2 independent investigators to evaluate eligibility, and any discrepancies were settled by discussion and consensus. First, the titles and abstracts of searched studies were screened. Then, full papers were reviewed to examine whether each study met the following criteria:

1. randomized controlled trial;
2. types of participants must be patients with symptomatic diagnosed lumbar degenerative diseases;
3. studies using total disc replacement and fusion for the treatment of lumbar degenerative diseases.

When multiple time points were reported either in one particular report of a study or over the course of several articles from the same study, the longest follow-up period on treatment was considered in our article. If overlapping subject populations were enrolled in different reports, the one of longest follow-up period was selected for inclusion. Full texts of all references were available.

### 2.3 Exclusion criteria

The excluded studies were excluded due to the following reasons:

1. studies does not conform to the above criteria;
2. treatment measures include other methods besides total disc replacement and lumbar fusion;
3. studies were in the form of letters, abstracts, reviews, or comments;
4. studies were impossible to extract relevant data.

### 2.4 Data extraction

The following data were independently extracted by 2 authors: the name of first author, year of publication, country, number of patients under total disc replacement and lumbar fusion, sample size, age, gender of patients, follow-up duration. When relevant data had not been reported, we contacted the authors by email or in other ways to attempt to obtain the missing information.

### 2.5 Quality assessment

We assessed the risk of bias of RCTs in this review using the Cochrane Collaboration Risk of Bias Tool. And risk of bias was assessed according to the Cochrane Handbook.^[18]^ For each included study, each type of bias was rated as high, low, or unclear and entered into the risk of bias table. 4 review authors, 2 with methodological expertise and 2 with content expertise, independently assessed the risk of bias of the included studies. The review authors resolved any disagreements by discussion, including input from a third independent review author if required.

### 2.6 Outcome measures

Visual analog scale (VAS) and Oswestry disability index (ODI) were the main outcomes, and the secondary outcomes were operation time, duration of hospitalization, blood loss, complications, patient satisfaction, work status, over successful, reoperation rate, ODI successful, device successful, and consumption of analgesics.

### 2.7 Grading the quality of evidence

The Grading of Recommendations Assessment, Development, and Evaluation (GRADE) method was used to assess the quality of the evidence for each outcome of meta-analysis. Levels of quality of evidence recommended by the GRADE Working Group were defined as high (++++), moderate (+++), low (++), and very low (+).^[19]^ The judgments were based on risk of bias, inconsistency, indirectness, imprecision, and publication bias.^[22]^ We operated on this web page: https://gradepro.org/.

### 2.8 Data synthesis and statistical analysis

The outcomes of interest include dichotomous data and continuous variables. Dichotomous data were expressed as the risk ratio (RR) and mean difference (MD) was used to assess the difference in the continuous outcomes between the groups. Also, standardized mean difference (SMD) was chosen if clinical outcome was the same but measured using different methods in the different trials. Its corresponding 95% confidence interval (CI) for each parameter was computed in total disc replacement-treated versus fusion-treated. Statistical heterogeneity across included studies was examined by the Q test and I^2^ statistic.^[50]^ An if *P* ≤ .1 and I^2^ ≥ 50% signified the possibility of statistical heterogeneity, and the random-effects model was chosen for the computation of MD or SMD with its corresponding 95% CI. Otherwise, no obvious heterogeneity (*P* > .1 and I^2^ < 50%) was considered to have occurred in the included studies, and the fixed-effects model was selected to generate the MD or SMD with its corresponding 95% CI. The forest plot for each parameter was constructed to illustrate the weight ratio of each incorporated study. All statistical analyses were carried out using the RevMan5.3 and STATA12.0 software. And the significance threshold was a 2-sided *P* < .05.

## 3 Results

### 3.1 Literature search and study sample characteristics

**Figure 1:**
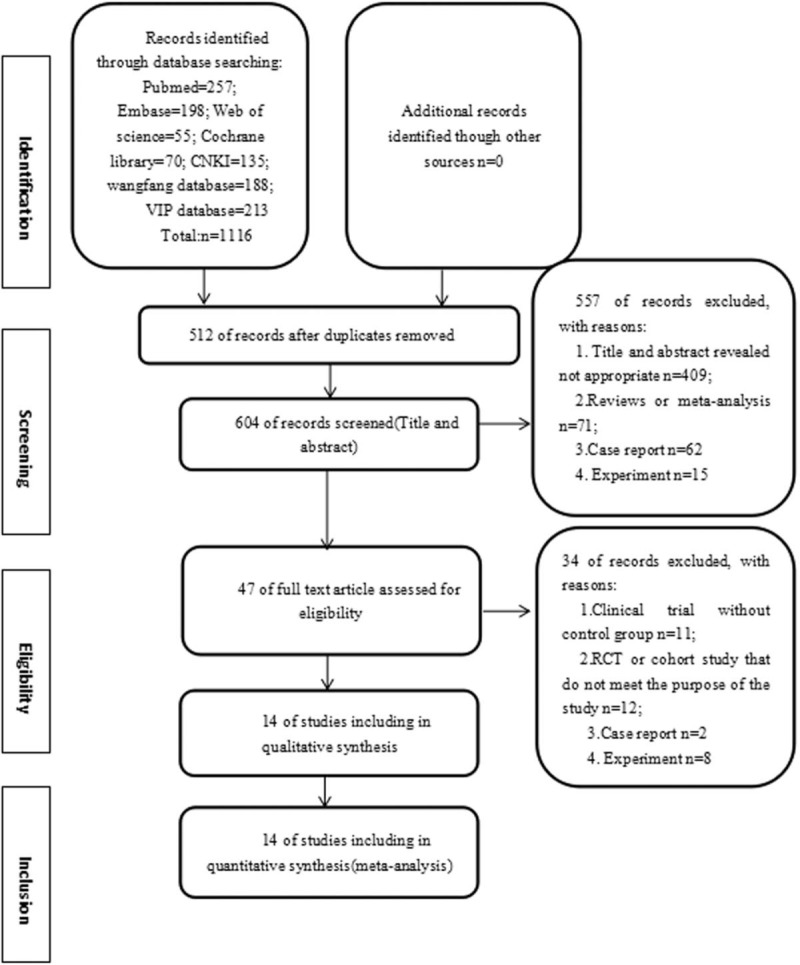
Flow diagram of literature search.

The search results are displayed in Figure 1. The primary searches identified a total of 1116 references using the outlined literature search strategy. Of these, 512 references were repeated literature in different databases and were excluded. According to the inclusion and exclusion criteria, 557 articles were excluded after reading the title and summaries. Then, after a detailed evaluation of full text, an additional 34 references were excluded. Among these, 11 trial was excluded because these are clinical trials lack to a control group. 12 studies were non-randomized controlled trials or quasi-randomized control trials, 2 case reports were excluded. 8 studies were excluded because these belong to the field of basic researches. Finally, 14 RCTs^[23–35,44]^ were included in the systematic review.

The characteristics of the included trials are summarized in Table 1. One trial^[35]^ was published in French and the rest were published in English. In this systematic review, a total of 1890 participants with lumbar degenerative diseases were involved. The trial sample size ranged from 32 to 577 participants. The type of artificial disc is one of 5 following devices: CHSRITE, ProDisc-L, ProDisc-II, MAVERICK and FlexiCore. And the control group included anterior fusion, posterior fusion and circumferential fusion. The intervention period is reported between 6 months to 5 years. Baseline imbalance was not found in the demographic characteristics or the outcomes between the study groups.

**Table 1:**
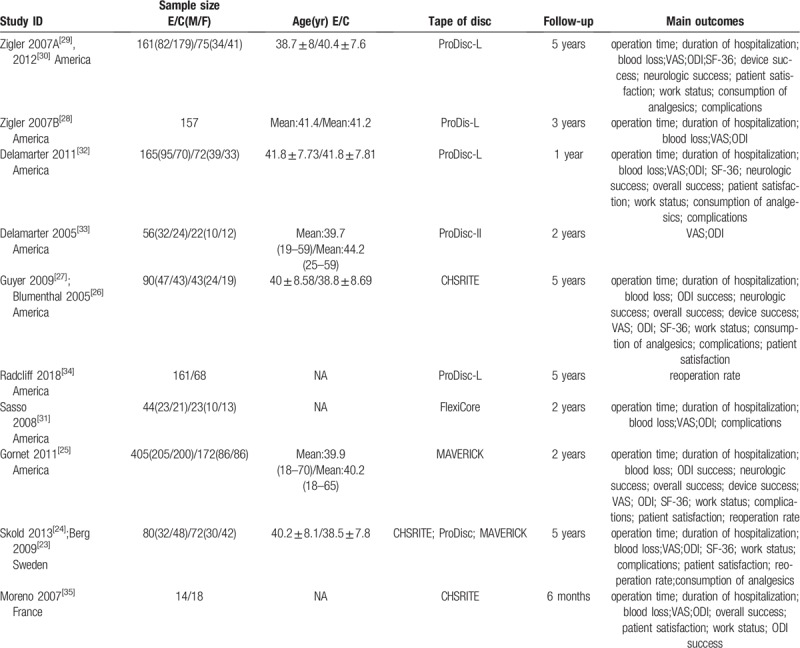
Basic characteristics of the included trials.

**Figure 2:**
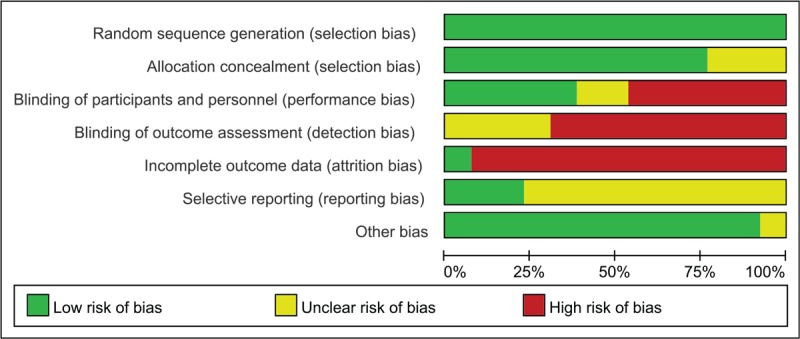
Risk of bias graph.

Figure 2 shows the graph of methodological quality. In the included studies, all trials described methods of randomization. The remaining 9 trials^[23–27,31,33–35]^ indicated “randomly allocating”. Five trials^[23–25,32,33]^ mentioned it use the blind method of participants. Twelve trials^[23–34]^ reported participant losses. Four trials^[25,32,34,35]^ have clinical trial registration. Selective reporting for other studies was difficult to assess, and trial protocols were unavailable. Ten studies^[23–25,28–30,32–35]^ found no significant other bias.

### 3.2 Meta-analysis results

#### 3.2.1 VAS

VAS was report in 12 studies.^[23–33,35]^ Among these, 4 articles^[23,24,29,30]^ were reported at different stages of the same study, both of which had a record of VAS, and selected Skold et al^[24]^ and Zigler et al study^[30]^ with long follow-up were used for analysis. Besides, 5 references^[26–28,31,33]^ data cannot be completely extracted, so the results can only be displayed in method of description. So 5 studies^[24,25,30,32,35]^ are included in the meta-analysis. The insignificant heterogeneity between trials was observed (*P* > .1, I^2^ = 0%), and therefore a fixed-effects model was used for statistical analysis (Fig. S1). Results from the pooled analysis indicated that there was a significant differences in improving VAS in favor of the total disc replacement (SMD = −0.206; 95% CI: −0.326 to −0.085; *P* = .001). In the study of Blumenthal et al,^[26]^ At 6 weeks, 3 months, 6 months and 12 months after surgery, disc replacement was more effective than spinal fusion in relieving pain symptoms, but 24 months later, there was no significant difference between the 2 methods. And there was no statistical difference between this 2 groups in terms of VAS at 5-year postoperative time point in Guyer study.^[27]^ Zigler et al^[28]^ result was although mean total disc replacement VAS scores were less than the fusion scores at each follow-up period, differences were not statistically significant. Sasso^[31]^ observed total disc replacement delivered improvements in pain similar to fusion. At last, Delamarter et al^[33]^ study found patients who received a disc replacement had a significant decrease in VAS score as early as 6 weeks and 3 months compared with fusion patients. However, the disc replacement patients continued to show more improvement than fusion patients, the difference was not significant.

#### 3.2.2 ODI

Twelve trials^[23–33,35]^ reported ODI as an outcome in the groups, similar to the outcome indicator --VAS, as for articles for different stages of the same study, Skold study^[24]^ and Zigler study^[30]^ were selected to analysis due to the long follow-up periods. Also, 5 studies^[26,27,28,31,33]^ were reviewed owing to these data cannot be completely extracted. So the rest studies^[24,25,30,32,35]^ were analyzed and the insignificant heterogeneity between trials was observed (*P* > .1, I^2^ = 0%), and therefore a fixed-effects model was used for statistical analysis (Fig. S2). Results from the pooled analysis indicated that there was a significant differences in improving VAS in favor of the total disc replacement (SMD = −0.276; 95% CI: −0.4 to −0.152; *P* < .0001). In the study of Blumenthal et al,^[26]^ ODI of the disc replacement group improved better than that of the lumbar fusion group after operation, but after 24 months, there was no significant difference between the two groups. Guyer et al^[27]^ study demonstrated there are no statistical difference between the groups in terms of ODI scores at the 2 and 5-year postoperative time points. Similar results with the VAS results, the total disc replacement ODI scores were less than the fusion scores at each follow-up period, the difference was statistically significant only at 3-month follow-up in Zigler study.^[28]^ Sasso et al^[31]^ observed total disc replacement delivered improvements in ODI scores similar to lumbar fusion. Delamarter et al^[33]^ yielded the result that disc replacement patients had significantly more reduction as early as 3 months, at 6 months and later up to 2 years, disc replacement and fusion patients had similar scores on ODI.

#### 3.2.3 Intraoperative conditions (operation time; blood loss; duration of hospitalization)

(1)The intraoperative conditions, including operation time, blood loss, duration of hospitalization, was shown in Figure S3,S4,S5, the results of the 9 trials^[23–28,30,32,35]^ were included, Berg et al^[23]^ and Skold et al^[24]^ studies had the same intraoperative data, so meta-analysis included only Berg findings.^[23]^ For the same reason, in the studies of Zigle et al^[29]^ and Zigle et al,^[30]^ the result of Zigle et al^[30]^ was chosen. Also, Blumenthal et al^[26]^ and Guyeret et al^[27]^ are different stages of the same study, Guyeret study^[27]^ was selected to analysis due to the long follow-up periods. Zigle et al^[28]^ research lacks of standard deviation, so data cannot be extracted for meta-analysis, and the results are displayed in Table 2. And the operation time, blood loss and duration of hospitalization of total disc replacement were lower than those of lumbar fusion surgery. Finally, 5 studies^[23,25,27,29,32]^ were analyzed. First, in term of operation time, these trials exhibited significant heterogeneity (*P* < .1, I^2^ = 98.3%), as shown in Figure S3. And accordingly, a random-effects model was used for statistical analysis. The meta=analysis of 5 trials revealed that total disc replacement group have a statistically significant decrease in operation time (SMD= −0.294; 95% CI: −0.416 to −0.173; Z = 4.75; *P* < .00001). Second, in regard to comparison of the bleeding volume, there is no difference between the 2 methods of operation (SMD = −0.077; 95% CI: −0.041 to 0.194; *P* = .2). Third, There was statistically significant difference between the total disc replacement therapy and lumbar fusion therapy in duration of hospitalization. The meta-analysis from the 5 independent trials revealed total disc replacement can significant reduce hospital stay (SMD = −0.447; 95% CI: −0.565 to −0.33; *P* < .00001).

**Table 2:**
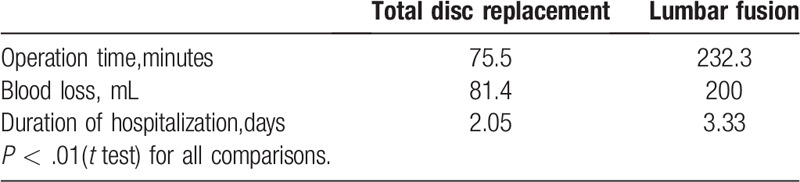
Zigle et al^[28]^ results.

#### 3.2.4 SF-36

SF-36 was report in 5 studies.^[23,24,25,30,32]^ Berg et al^[23]^ and Skold et al^[24]^ studies belong to the same randomized controlled trial. So Skold study was analyzed. The insignificant heterogeneity between trials was observed (*P* > .1, I^2^ = 0%), and therefore a fixed-effects model was used for statistical analysis (Fig. S6). The meta analysis from the 4 independent trials demonstrated that participants treated with total disc replacement therapy improving more significantly than participants treated with lumbar fusion therapy (SMD = 0.283; 95% CI: 0.157 to 0.409; *P* < .0001).

#### 3.2.5 Consumption of analgesics

Of the 4 trials^[24,26,30,32]^ that documented consumption of analgesics, as shown in Figure S7. These trials exhibited significant heterogeneity (*P* < .1, I^2^ = 82.6%). And accordingly, a random-effects model was used for statistical analysis. The meta-analysis of 4 trials revealed that there are not statistically significant in consumption of analgesics (RR = 0.909; 95% CI: 0.633 to 1.306; *P* = 0.606).

#### 3.2.6 Patient satisfaction

Data regarding the patient satisfaction are shown in Figure S8. The results of 7 trials^[23–26,30,32,35]^ were included, Berg et al^[23]^ and Skold et al^[24]^ studies were the same trials, so meta-analysis included only Skold findings^[23]^ due to the long follow-up periods. Thus, the results revealed a significant difference in participants treated with total disc replacement and those treated with lumbar fusion, with total disc replacement therapy being favored (RR = 1.183; 95% CI: 1.106 to 1.264; *P* < .0001;I^2^ = 8.4%).

#### 3.2.7 Work status

Nine trials^[23–27,29,30,32,35]^ used work status as an outcome. The same reason as before, Skold study,^[24]^ Guyer study^[27]^ and Zigler et al^[30]^ with long follow-up were used for analysis rather than Berg study^[23]^, Blumenthal study^[26]^ and Zigler study.^[29]^ Significant heterogeneity between trials was observed (*P* < .1, I^2^ = 56.5%), so a rand-effects model was used for statistical analysis. However, there was no significant difference in work status between total disc replacement and lumbar fusion (RR = 0.968; 95% CI: 0.873 to 1.074; *P* = .543) (Fig. S9).

#### 3.2.8 Over success

A comparison of over success is shown in Figure S10. Five studies^[25,26,27,32,35]^ reported over success as an outcome. And Guyer et al^[27]^ and Blumenthal et al^[26]^ studies were the same trials, so meta-analysis included only Guyer result^[27]^ due to the long follow-up periods. Hence, pooled analysis indicated that over success improved significantly more in the total disc replacement group than lumbar fusion (RR = 1.272; 95% CI: 1.109 to 1.458; *P* = .001; I^2^ = 0).

#### 3.2.9 Neurologic success

Neurologic success data from 4 trials^[25,26,27,32]^ appear in Figure S11. Same as before, the meta-analysis included only Guyer result^[27]^ instead of Blumenthal et al^[26]^ finding due to the long follow-up periods. And comparisons of neurologic success with total disc replacement and lumbar fusion displayed insignificant heterogeneity (*P* > .1, I^2^ = 0) between studies. Thus, a fixed-effects model was used for analysis and there was no statistical difference between 2 methods for neurologic success variations (RR = 1.035; 95% CI: 0.979 to 1.093; *P* = .223), although this has a tendency to benefit the total disc replacement group.

#### 3.2.10 Reoperation rate

Seven trials^[23,24,25,29,30,32,34]^ assessed the incidence of reoperation of total disc replacement in comparison with lumbar fusion. Due to some articles are different stages of the same study, Berg study^[23]^ and Zigler study^[29]^ were not to be analyzed due to the short follow-up periods. Thus, meta-analysis showed that there was a significant difference found in favor of the total disc replacement (RR = 0.534; 95% CI: 0.288 to 0.992; *P* = .047) (Fig. S12).

#### 3.2.11 ODI success

Six studies^[23,24,25,26,27,35]^ reported ODI success, but Berg study^[23]^ and Blumenthal study^[26]^ were not to be analyzed due to the repetition with Skold study^[24]^ and Guyer study.^[27]^ So, the rest studies were analyzed and a fixed-effects model was used for statistical analysis according to the low heterogeneity (*P* > .1, I^2^ = 0). The results displayed that significant differences in favor of the total disc replacement (RR = 1.116; 95% CI: 1.025 to 1.216; *P* = .011) (Fig. S13).

#### 3.2.12 Device success

With regard to the device success, there were 3 studies^[26,27,30]^ eligible for this analysis. The meta-analysis included only Guyer result^[27]^ instead of Blumenthal et al^[26]^ finding due to the long follow-up periods. And a small degree of heterogeneity was found in the analysis (*P* > .1, I^2^ = 0), thus, a fixed-effects model was selected for construction of forest plots. And the results indicated that no significant difference was detected in device success (RR = 1.055; 95% CI: 0.987 to 1.128; *P* = .115) (Fig. S14), although this has a tendency to benefit the total disc replacement.

#### 3.2.13 Complications

In term of complications, there were 8 eligible studies^[23–26,29–32]^ for this analysis (Fig. S15). For the same study, only the longest follow-up trial was selected. So Skold study^[24]^ and Zigler et al^[30]^ were included in the analysis. Thus, the meta analysis from the 6 independent trials demonstrated that participants treated with lumbar fusion therapy leading to more complications than participants treated with total disc replacement therapy (RR = 0.437, 95% CI: 0.282 to 0.678, *P* < .0001).

#### 3.2.14 Charge analysis

Of 14 studies, only 1 study^[44]^ makes charge analysis. A total of 53 patients were included in the study, including 36 in total disc replacement group and 17 in fusion group. For patients with 1-level disease, the charge analysis shows significant difference between total disc replacement and fusion group. The mean total charge for the total disc replacement group was $35,592 versus $46,280 for the fusion group (*P* = .0018). For patients with 2-level disease, the charge analysis shows no significant difference between total disc replacement group and fusion group. As shown in the Table 4, the mean total charge is $55,524 in total disc replacement group and $56,823 in fusion group(*P* = .55).

**Table 3:**
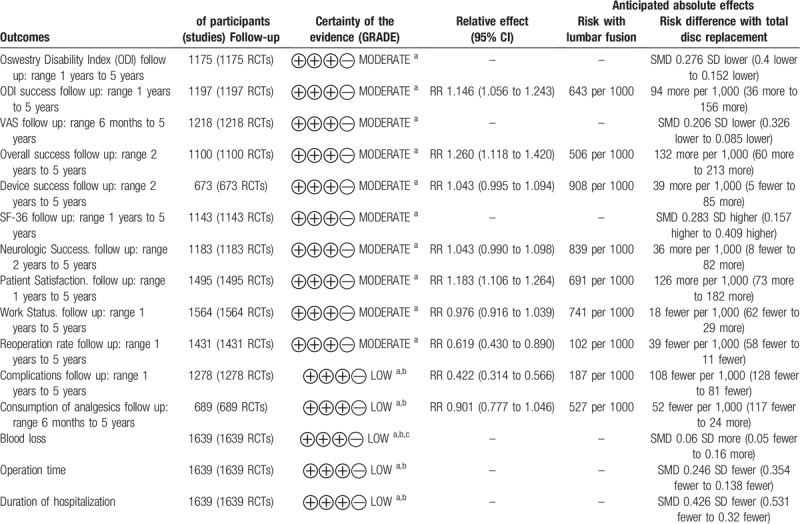
Summary of the evidence for each outcome.

**Table 4:**
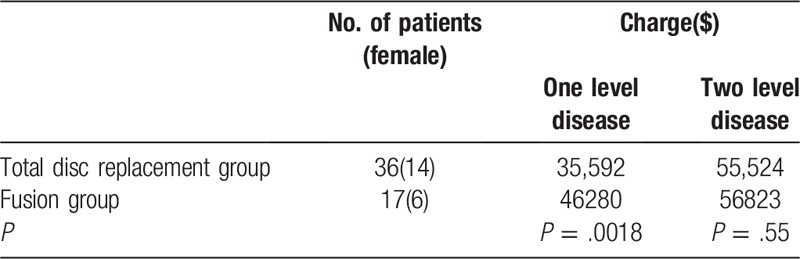
Charge analysis about total disc replacement and lumbar fusion.

#### 3.2.15 Publication bias

The funnel plot of each meta-analysis is provided in Appendix S2. We found that only this outcome of device success (*P* = .019) had a certain publication bias by using Egger regression test.

#### 3.2.16 Grade

The GRADE level of evidence is low for consumption of analgesics, blood loss, operation time, duration of hospitalization, but moderate for the rest outcomes. Table 3 shows the GRADE evidence profiles. The main reasons for a deceasing level were high dropout rate, high heterogeneity and intersecting invalid lines.

## 4 Discussion

Lumbar degenerative disease is essentially characterized by lumbar or/and leg pain with or without walking difficulties due to some specific situations, such as narrowing of spinal canal, prominent discs, degenerative disc disease, arthropathy and spondylolisthesis.^[36]^ Most of the time surgical treatment is necessary to relieve the symptoms, and spinal fusion is deemed to the gold standard for treatment of spinal degenerative disease.^[37,38]^ More and more studies have described the weakness after the fusion operation, including adjacent segment degeneration (ASD) and acquired spinal instability.^[37]^ However, these complications can be effectively avoided by performing a total disc replacement surgery, as an alternative technique, which is made up of bearing surfaces designed to accommodate load without breaking, to reduce friction and wear and to keep range of motion as long as possible.^[39–41]^

Our systematic review and meta-analysis found that disc replacement is superior to lumbar fusion in many respects, including ODI, VAS, SF-36, patient satisfaction, overall success, reoperation rate, ODI successful. In addition, postoperative complications of disc replacement surgery are also less than lumbar fusion. Therefore, the superiority of total disc replacement over lumbar fusion may be partially explained by the relatively simple surgical procedure and decreased postsurgical complications in the disc replacement group. As is known to us all, disc replacement surgery is usually done through anterior approach. However, lumbar fusion still requires bone grafting from other body parts or the use of allogeneic bone for interbody or posterolateral fusion. Thus, this adds a lot of surgical procedures. In particular, if a combined anterior and posterior lumbar fusion surgery is performed, more surgical steps are needed. So, as for intraoperative conditions, total disc replacement can significant reduce operation time and hospital stay. Although there is no difference for bleeding volume between the two methods of operation, the trend of bleeding volume of lumbar disc replacement surgery is less than that of lumbar fusion surgery is very obvious. The operation of intervertebral disc replacement is simple, so there are fewer complications after operation, so the clinical effect after operation is more remarkable. Meanwhile, less hospital stay is available. Besides, the device of total disc replacement is designed in a motion preservation technology. So the use of lumbar disc replacement in anticipation of minimizing the development of adjacent segment disease.^[39,42,43]^ These factors also contribute to improving the functional status of the lumbar spine for patients who treated by total disc replacement operation. Moreover, with regard to consumption of analgesics, neurologic success and device success, our meta-analysis results indicated that no significant difference was detected between 2 operative methods. As we all know, the main purpose of narcotic use is to relieve the pain of the incision after operation, so the degree of incision pain may be similar between the two surgical methods. Also, Since both methods improve the symptoms of lumbar spinal stenosis, this may explain that there is no difference in the effect of the 2 treatments on nerve function. At last, device success was defined as the absence of any need for reoperation to modify or remove implants and no need for additional fixation.^[30]^ As shown in Figure S14, there is a tendency to benefit for the total disc replacement. The result of no difference between 2 methods for device success may be that few papers (only 2 papers) were included, and more research is needed to clarify this result. As for charge analysis, it shows significant difference between total disc replacement and fusion group for patients with 1-level disease, but insignificant difference for patients with 2-level disease. So, chargers with total disc replacement are significantly low compared with lumbar fusion in the 1-level patients.

Several previous meta-analyses which can be retrieved have reported on the same topic (Table 5). Compared with previous meta-analyses, the present meta-analysis has the following advantages. First, the present systematic review retrieved the latest literature and more databases and included more literature and participants. As the latest and most comprehensively updated meta-analysis, the present study contains a more comprehensive outcome indicator, which can more effectively evaluate the effectiveness and security of total disc replacement. For the main outcome index in this study, Visual analog scale (VAS) and Oswestry disability index (ODI), the present study showed significant improvement in the total disc replacement group after 5 years of follow-up, which is consistent with previous studies. For outcomes like ODI success, device success, neurologic success and SF-36, which the previous study did not report, only this study made analysis. For outcome reoperation rate, the present study changes the previous conclusion and show significant improvement in total disc replacement group. For outcomes like complications and duration of hospitalization, previous studies provided contradictory conclusions, but this study provided the most reliable conclusion by including the latest and most comprehensively updated literature. Second, the protocol of this study was registered on PROSPERO. As we all know, a registered protocol may increase the transparency and quality of meta-analysis. Third, we adopted the GRADE approach to assess the quality of evidence. Thus, the conclusions of this study can be clinically used and easily transferred to guidelines. Fourth, this is the first meta-analysis including cost analysis. It is more comprehensive to evaluate the advantages and disadvantages of the two surgical methods, which is of great significance to clinical practice.

**Table 5:**
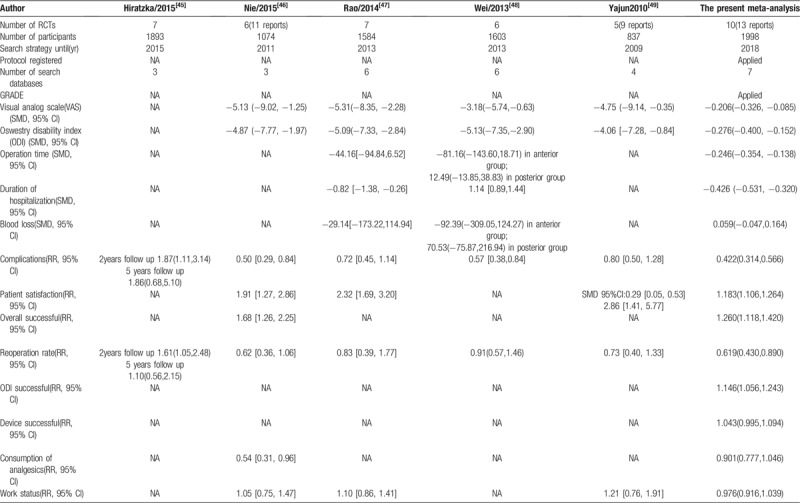
Comparison with other previous meta-analyses.

The present meta-analysis has the following limitations. First, some outcome indicators have significant heterogeneity, and we speculated that heterogeneity may come from these risk factors, such as different patient groups, different devices, different fixed tools, and various clinical settings, especially different medical centers and different surgical and anesthetic techniques. However, subgroup analysis cannot be performed because some outcomes just contain 2 to 3 literature. Second, because total disc replacement and lumbar fusion are the obvious operational manipulation, it could be difficultly blinded for doctors, this may lead to unavoidable performance bias. Third, some of articles failed to provide sufficient data, although we made an effect to obtain, including attempting to contact to correspondence author of articles.

## 5 Conclusion

This systematic review and meta-analysis suggests that total disc replacement surgery, compared to lumbar fusion surgery, significantly improved ODI, VAS, SF-36, patient satisfaction, overall success, reoperation rate, ODI successful, reduced operation time, shortened duration of hospitalization, decreased postsurgical complications. However, total disc replacement did not show a significant difference in terms of blood loss, consumption of analgesics, neurologic success and device success with lumbar fusion. And charges were significantly lower for total disc replacement compared with lumbar fusion in the 1-level patient group, while charges were similar in the 2-level group. Hence, total disc replacement is recommended to alleviate the pain of degenerative lumbar diseases, improve the state of lumbar function and the quality of life of patients, and provide a high level of security. Moreover, total disc replacement has better health economics benefits for 1-level patients.

## Author contributions

**Data curation:** Long Liang, Bing-bing Zhang.

**Formal analysis:** Long Liang, Bing-bing Zhang.

**Investigation:** Tao Zhu.

**Methodology:** Hai-jun Zhang, Zhi-guo Yuan, Tao Zhu.

**Software:** Hai-jun Zhang, Tao Zhu.

**Supervision:** Zhi-guo Yuan, Yanfei Chen.

**Validation:** Tao Zhu.

**Writing – original draft:** Dengyan Bai.

## Supplementary Material

Supplemental Digital Content
