# Supplementary material for: Total disc replacement versus fusion for lumbar degenerative diseases - a meta-analysis of randomized controlled trials
Source: Medicine (Baltimore). 2019 Jul 19;98(29):e16460. doi: 10.1097/MD.0000000000016460 (PMC6709089; doi:10.1097/MD.0000000000016460)
Supplement: Supplemental Digital Content [file medi-98-e16460-s001.pdf]

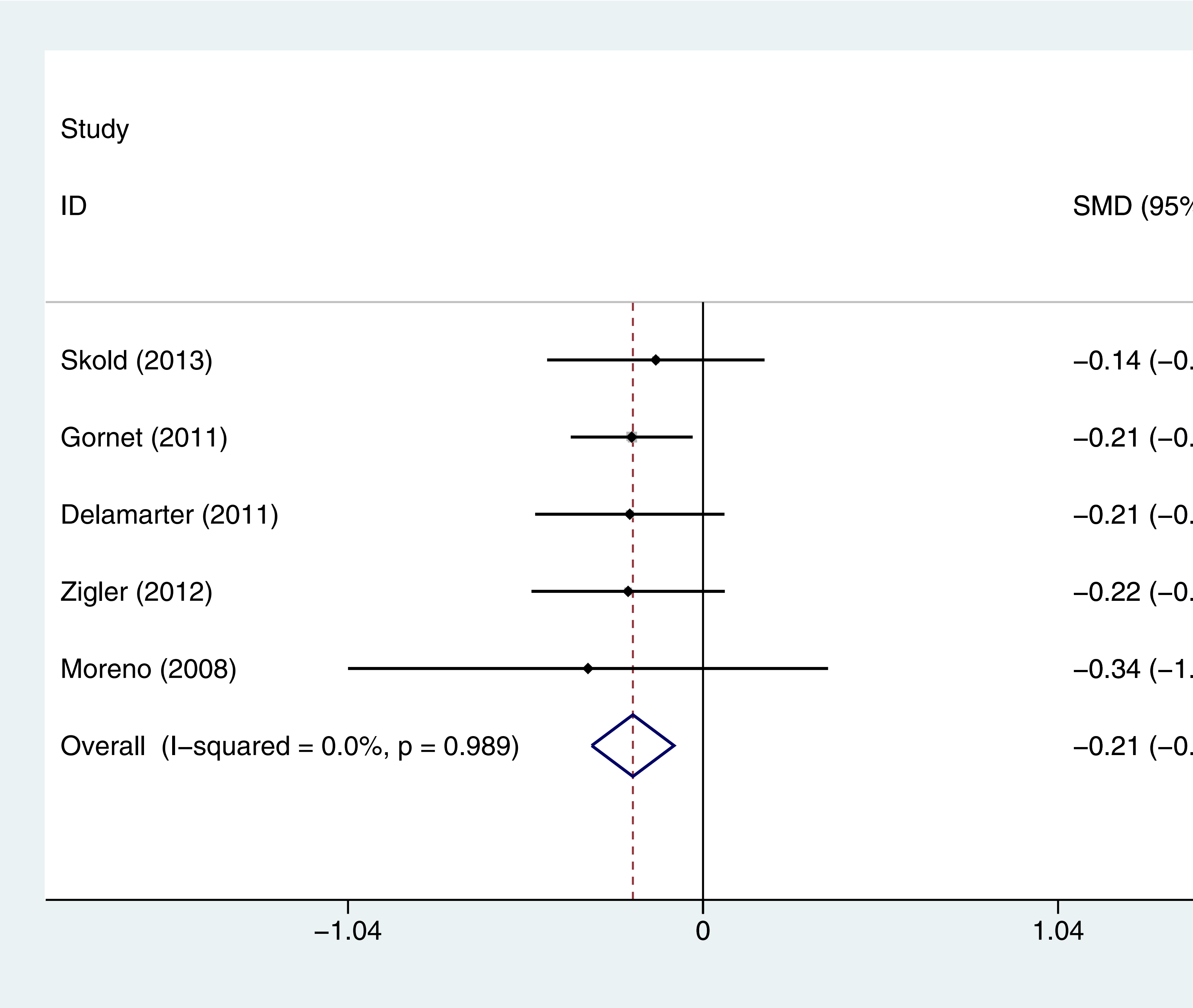

Study
ID
SMD (95%
Skold (2013)
−0.14 (−0.
Gornet (2011)
−0.21 (−0.
Delamarter (2011)
−0.21 (−0.
Zigler (2012)
−0.22 (−0.
Moreno (2008)
−0.34 (−1.
Overall (I−squared = 0.0%, p = 0.989)
−0.21 (−0.
−1.04
0
1.04

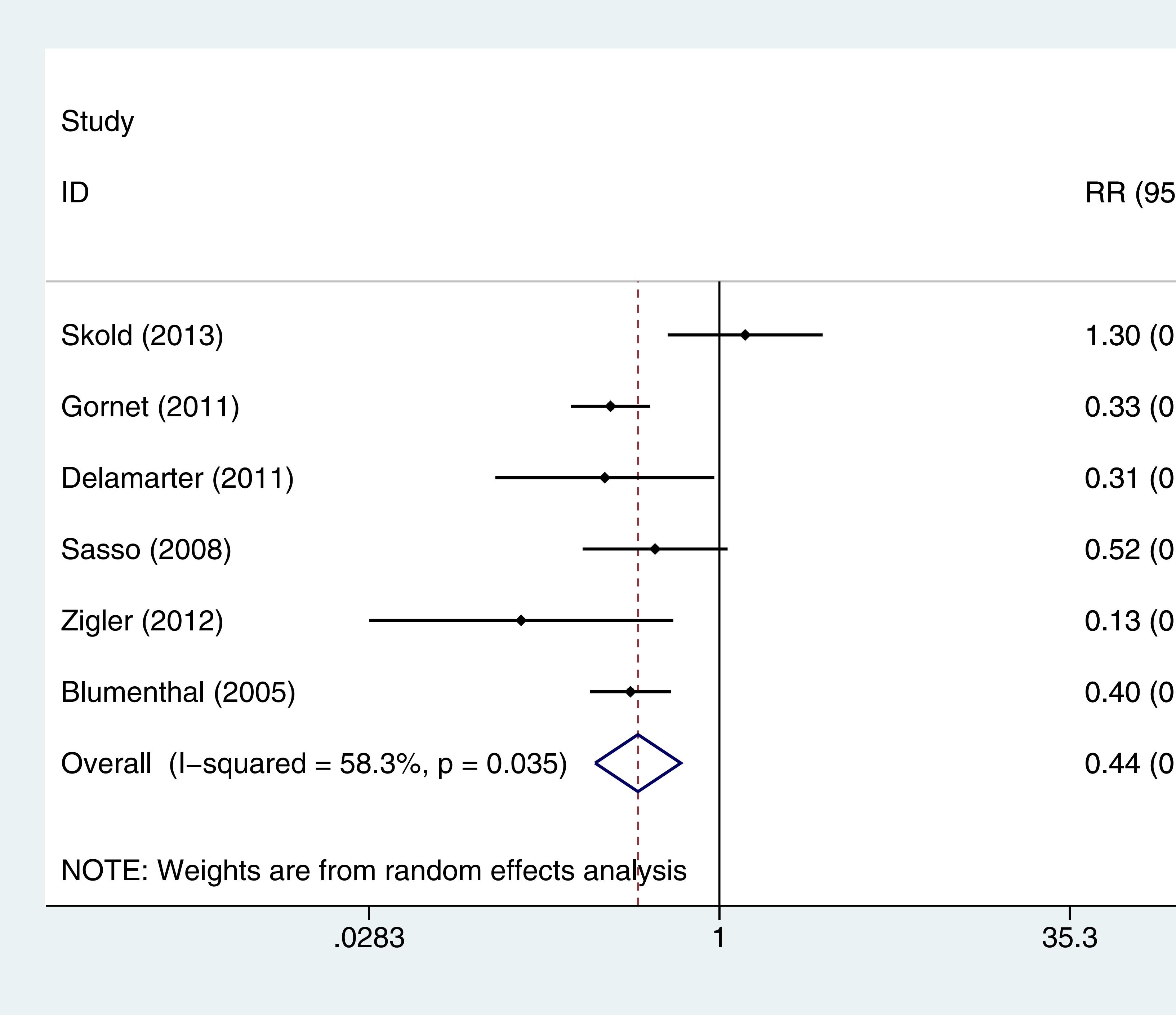
Study
ID
RR (95
Skold (2013)
1.30 (0
Gornet (2011)
0.33 (0
Delamarter (2011)
0.31 (0
Sasso (2008)
0.52 (0
Zigler (2012)
0.13 (0
Blumenthal (2005)
0.40 (0
Overall (I–squared = 58.3%, p = 0.035)
0.44 (0
NOTE: Weights are from random effects analysis
.0283
1
35.3

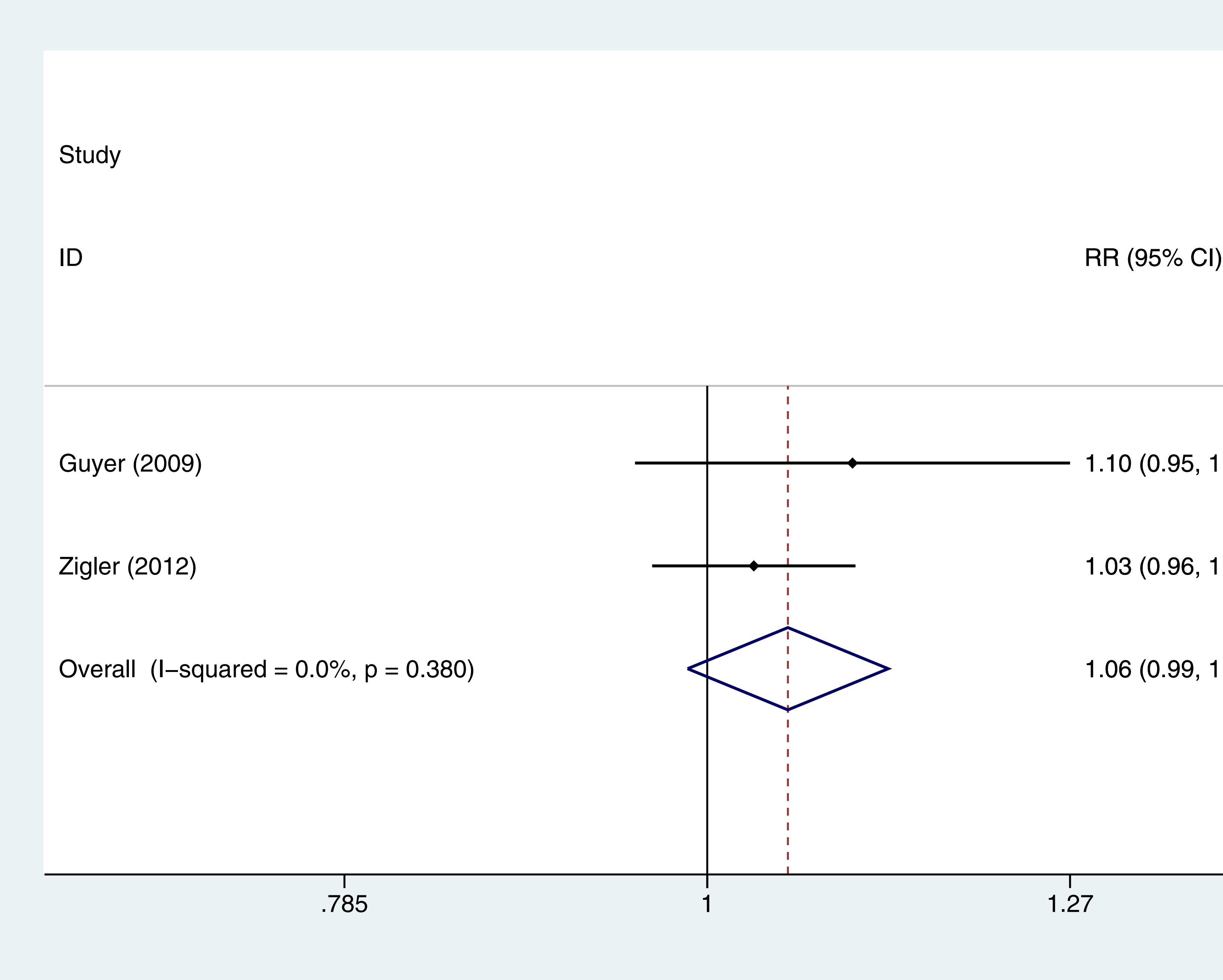

Study
ID
RR (95% CI)
Guyer (2009)
1.10 (0.95, 1
Zigler (2012)
1.03 (0.96, 1
Overall (I–squared = 0.0%, p = 0.380)
1.06 (0.99, 1
.785
1
1.27

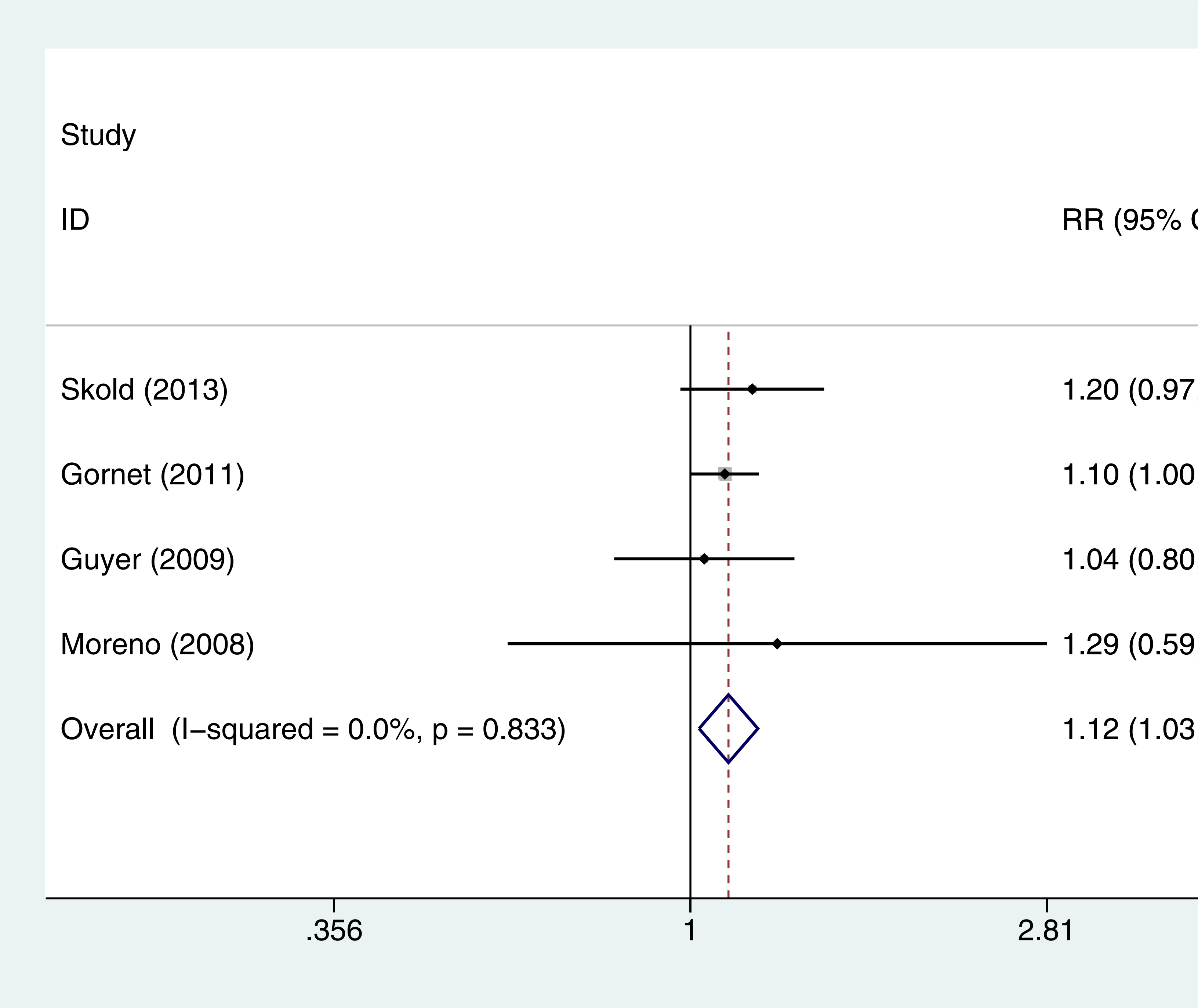

Study
ID
RR (95% C
Skold (2013)
1.20 (0.97,
Gornet (2011)
1.10 (1.00,
Guyer (2009)
1.04 (0.80,
Moreno (2008)
1.29 (0.59,
Overall (I–squared = 0.0%, p = 0.833)
1.12 (1.03,
.356
1
2.81

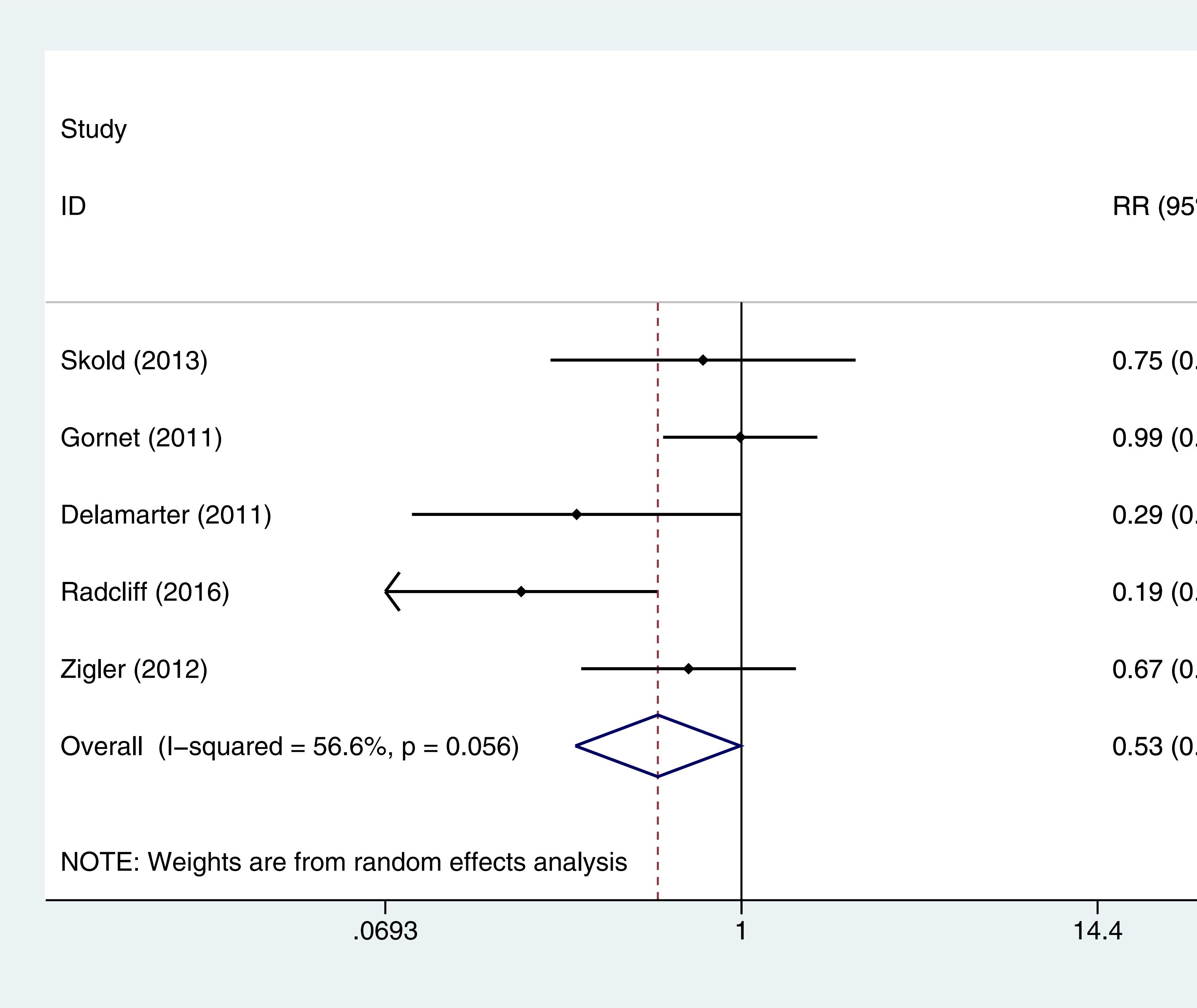

Study
ID
RR (95
Skold (2013)
Gornet (2011)
Delamarter (2011)
Radcliff (2016)
Zigler (2012)
Overall (I–squared = 56.6%, p = 0.056)
0.75 (0.
0.99 (0.
0.29 (0.
0.19 (0.
0.67 (0.
0.53 (0.
NOTE: Weights are from random effects analysis
.0693
1
14.4

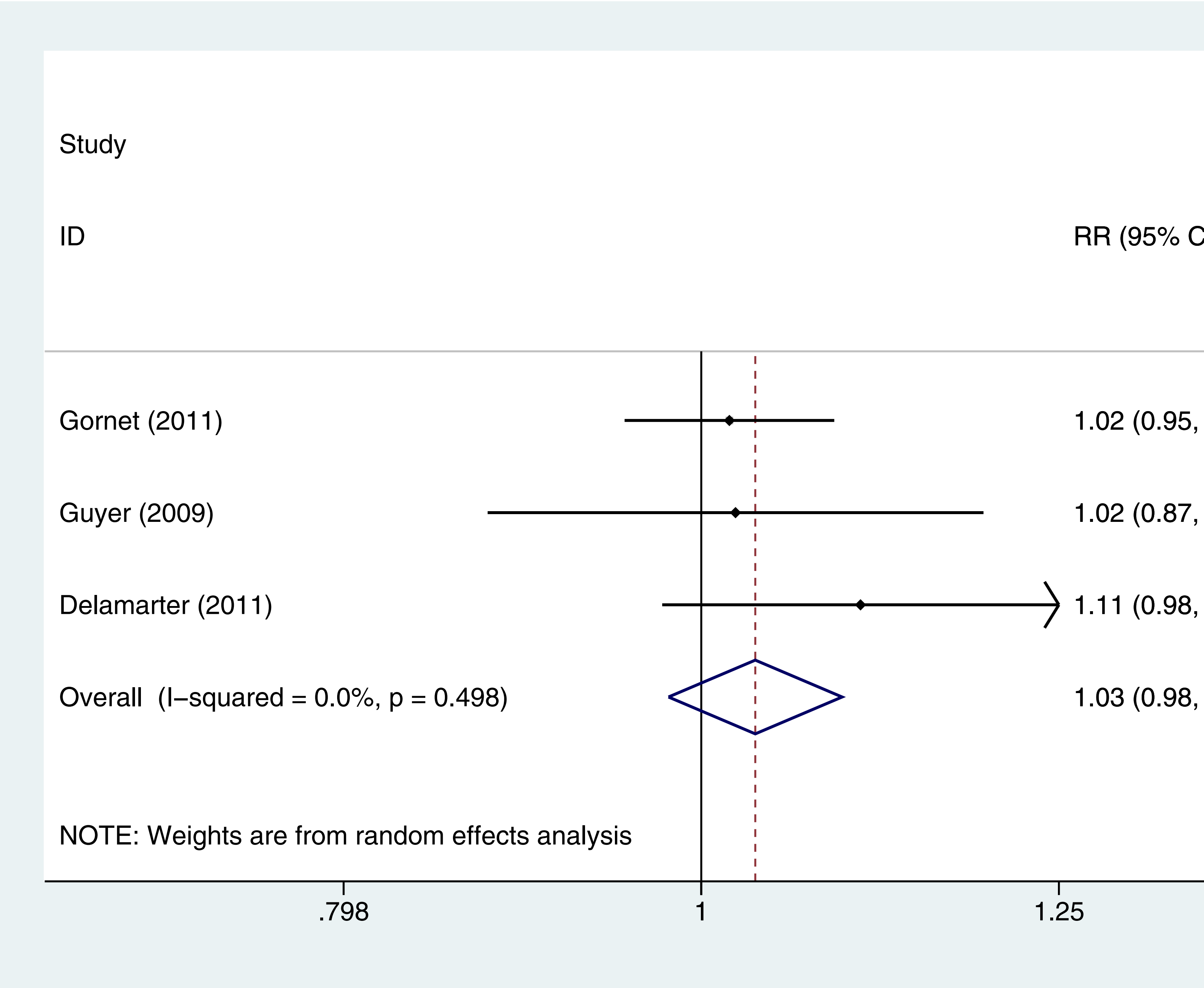
Study
ID
RR (95% C
Gornet (2011)
1.02 (0.95,
Guyer (2009)
1.02 (0.87,
Delamarter (2011)
1.11 (0.98,
Overall (I–squared = 0.0%, p = 0.498)
1.03 (0.98,
NOTE: Weights are from random effects analysis
.798
1
1.25

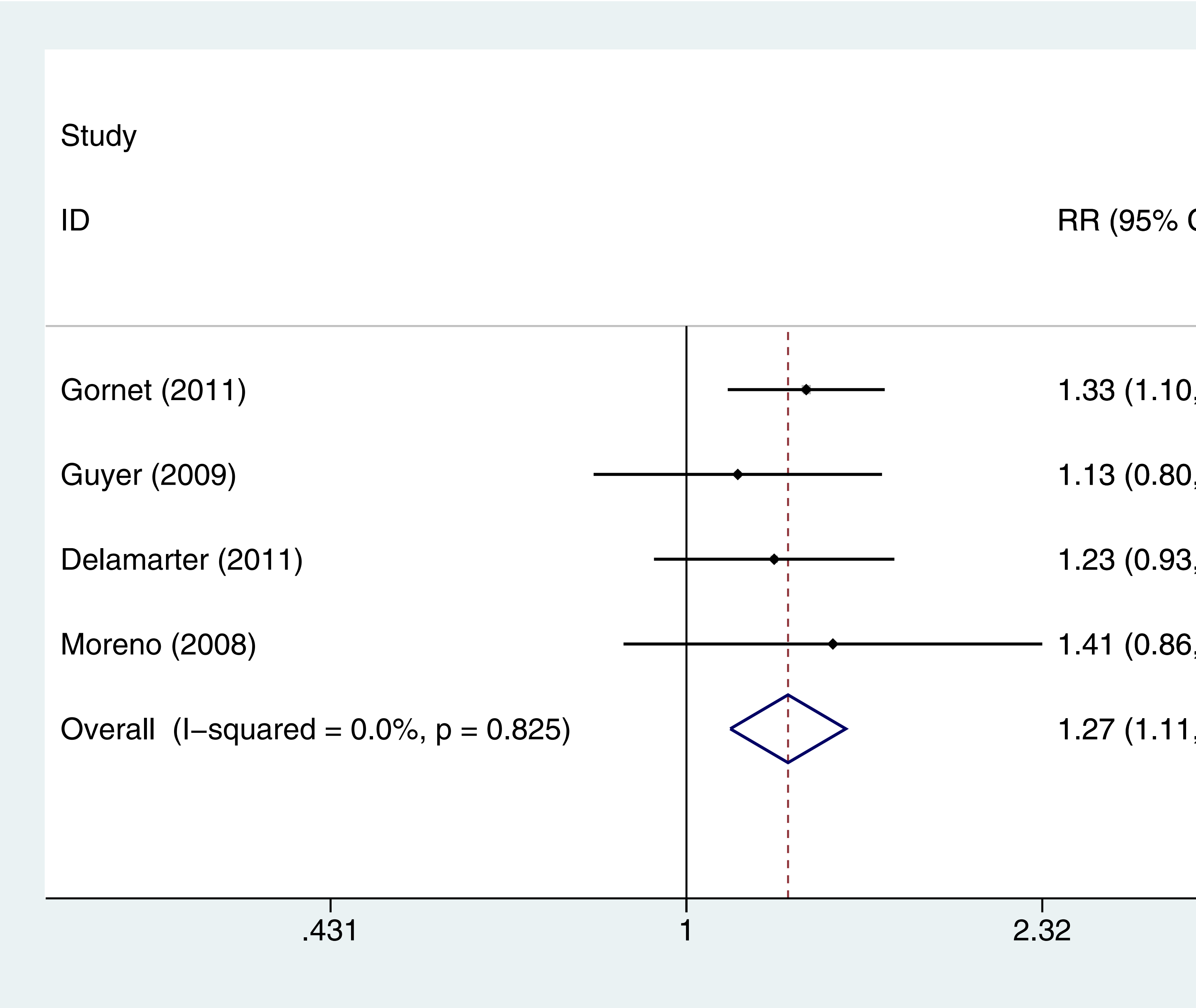

Study
ID
RR (95% C
Gornet (2011)
Guyer (2009)
Delamarter (2011)
Moreno (2008)
Overall (I–squared = 0.0%, p = 0.825)
1.33 (1.10,
1.13 (0.80,
1.23 (0.93,
1.41 (0.86,
1.27 (1.11,
.431
1
2.32

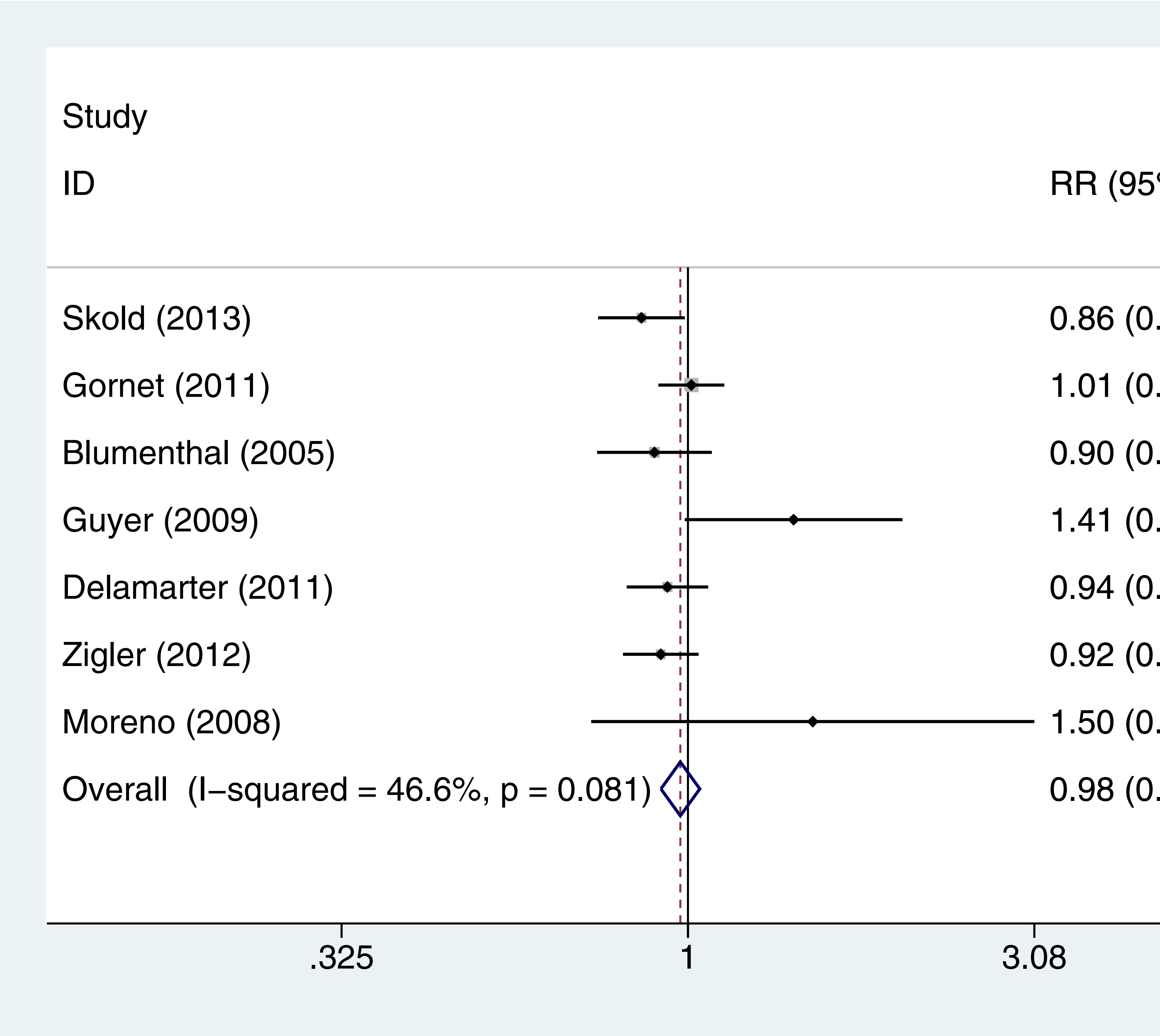

Study
ID
RR (95
Skold (2013)
0.86 (0.
Gornet (2011)
1.01 (0.
Blumenthal (2005)
0.90 (0.
Guyer (2009)
1.41 (0.
Delamarter (2011)
0.94 (0.
Zigler (2012)
0.92 (0.
Moreno (2008)
1.50 (0.
Overall (I–squared = 46.6%, p = 0.081)
0.98 (0.
.325
1
3.08

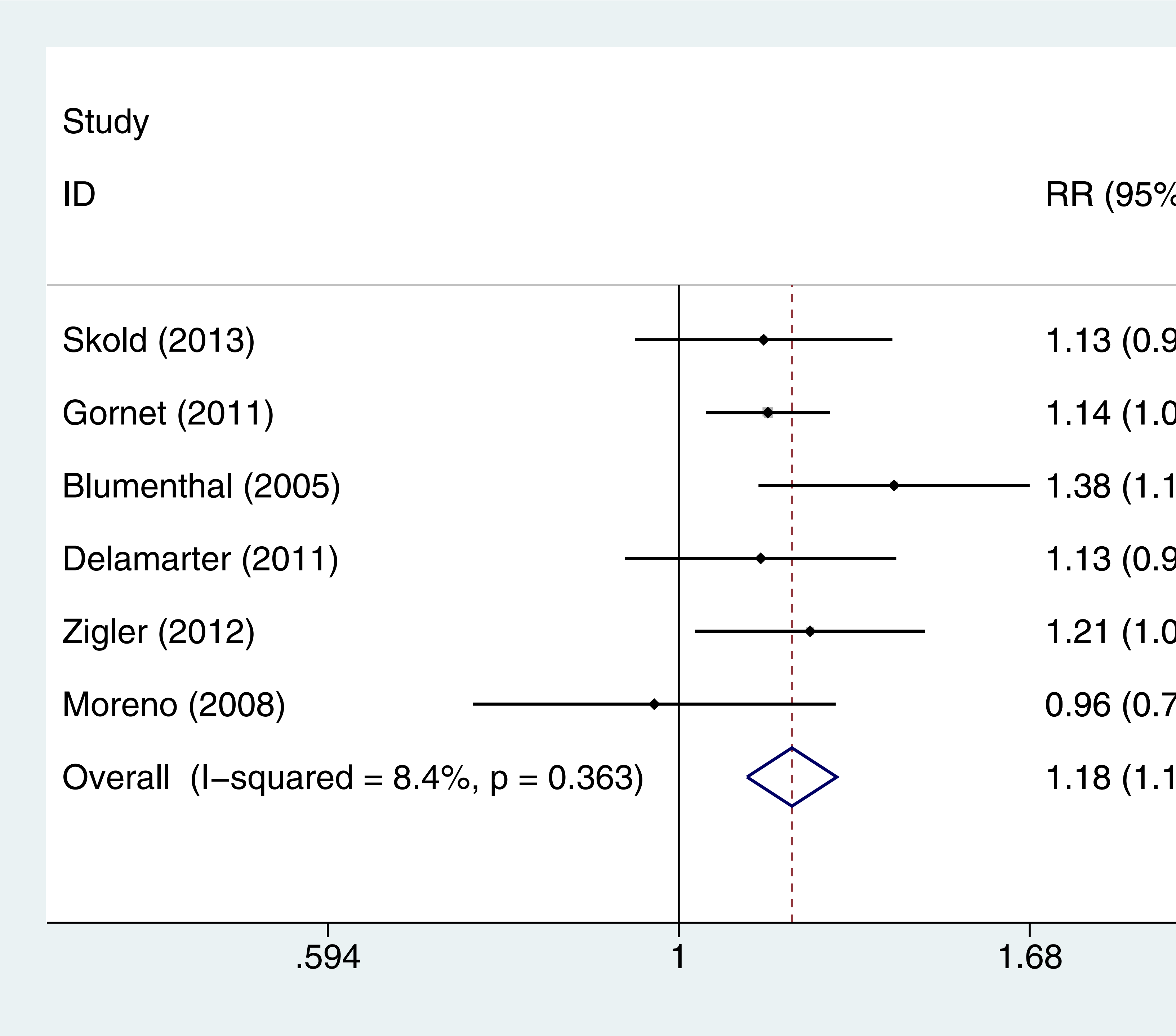
Study
ID
RR (95%
Skold (2013)
1.13 (0.9
Gornet (2011)
1.14 (1.0
Blumenthal (2005)
1.38 (1.1
Delamarter (2011)
1.13 (0.9
Zigler (2012)
1.21 (1.0
Moreno (2008)
0.96 (0.7
Overall (I–squared = 8.4%, p = 0.363)
1.18 (1.1
.594
1
1.68

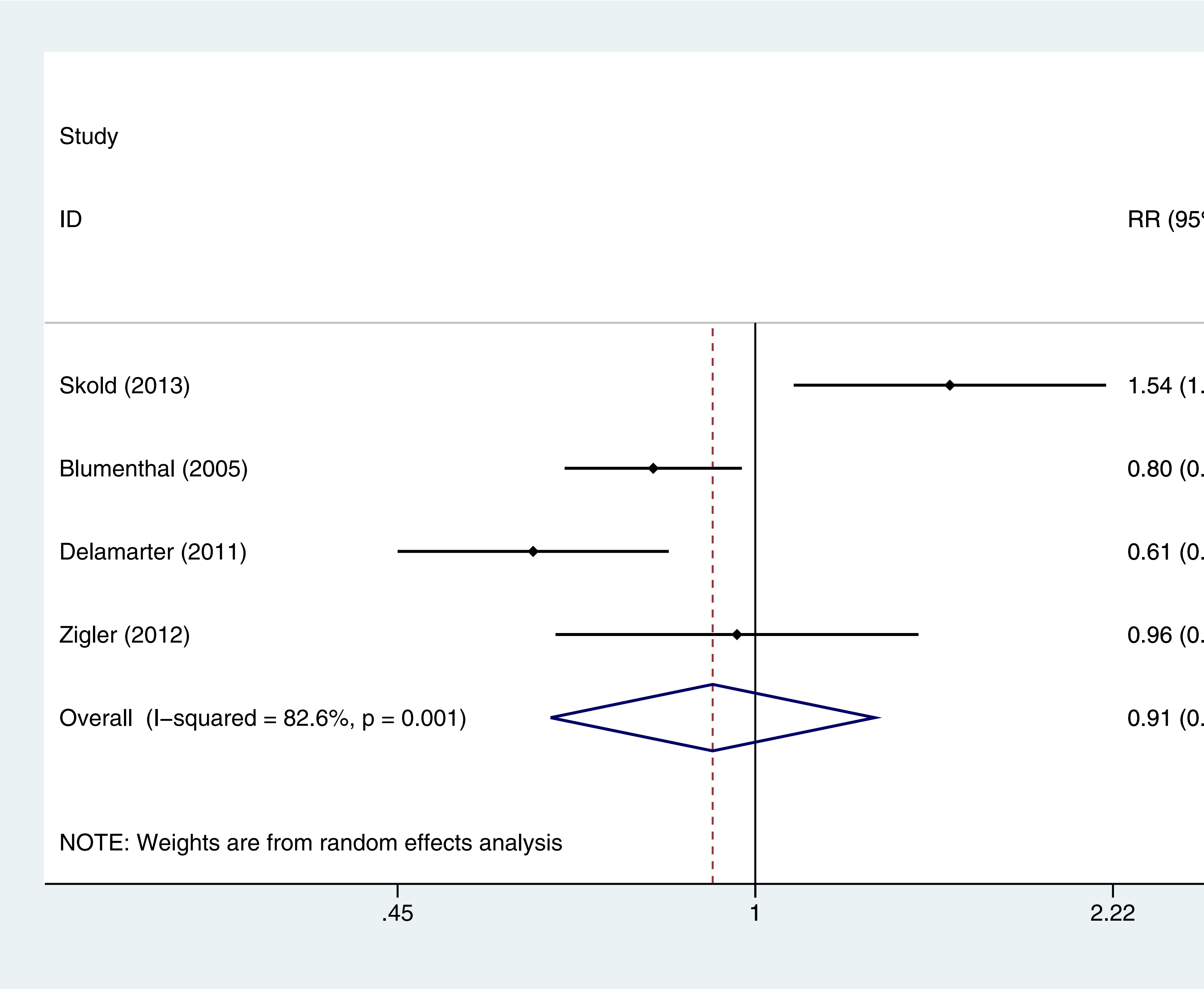

Study
ID
RR (95
Skold (2013)
1.54 (1.
Blumenthal (2005)
0.80 (0.
Delamarter (2011)
0.61 (0.
Zigler (2012)
0.96 (0.
Overall (I–squared = 82.6%, p = 0.001)
0.91 (0.
NOTE: Weights are from random effects analysis
.45
1
2.22

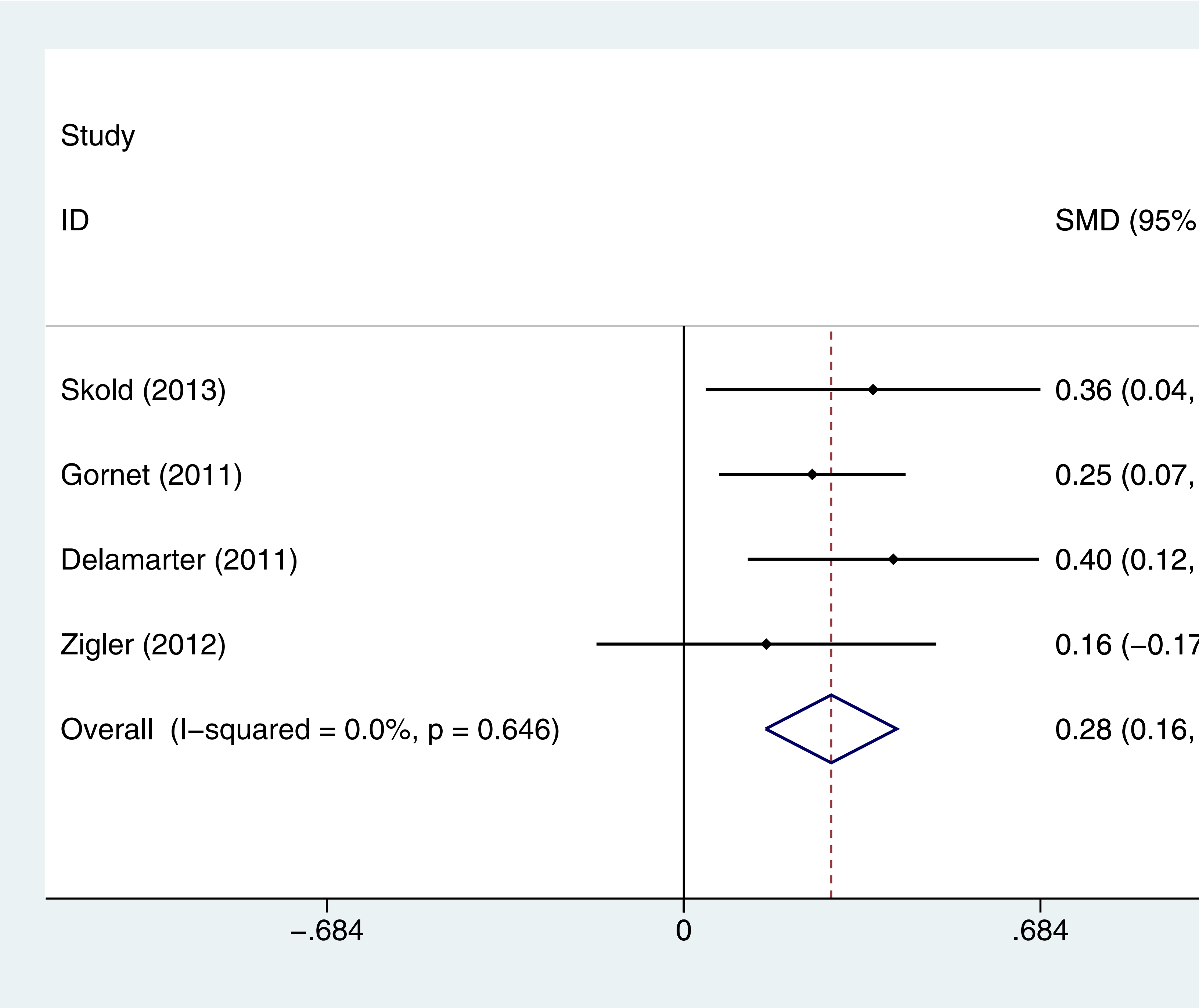

Study
ID
SMD (95%
Skold (2013)
0.36 (0.04,
Gornet (2011)
0.25 (0.07,
Delamarter (2011)
0.40 (0.12,
Zigler (2012)
0.16 (−0.17
Overall (I−squared = 0.0%, p = 0.646)
0.28 (0.16,
−.684
0
.684

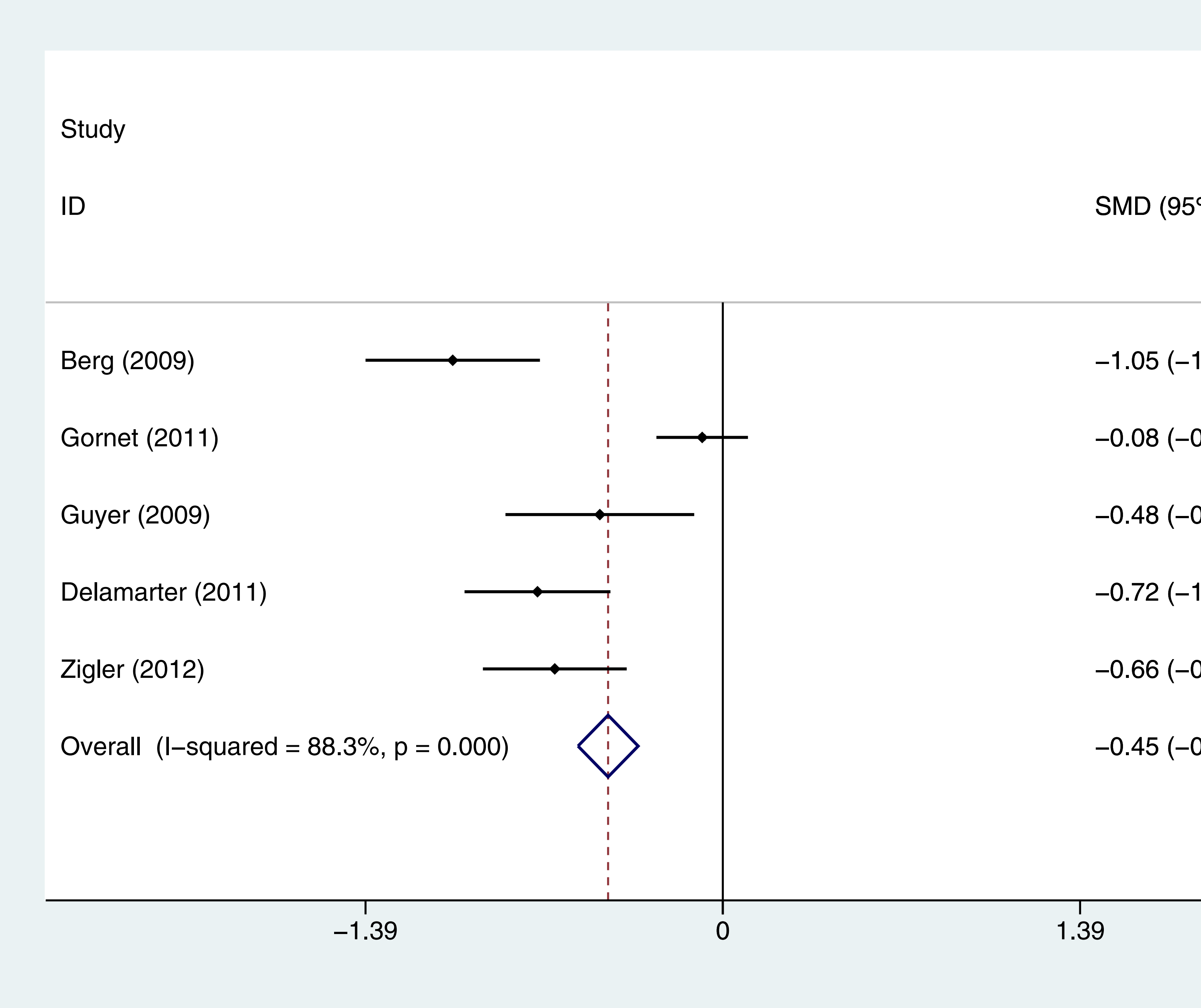

Study
ID
SMD
Berg (2009)
−1.05
Gornet (2011)
−0.08
Guyer (2009)
−0.48
Delamarter (2011)
−0.72
Zigler (2012)
−0.66
Overall (I−squared = 88.3%, p = 0.000)
−0.45
−1.39
0
1.39

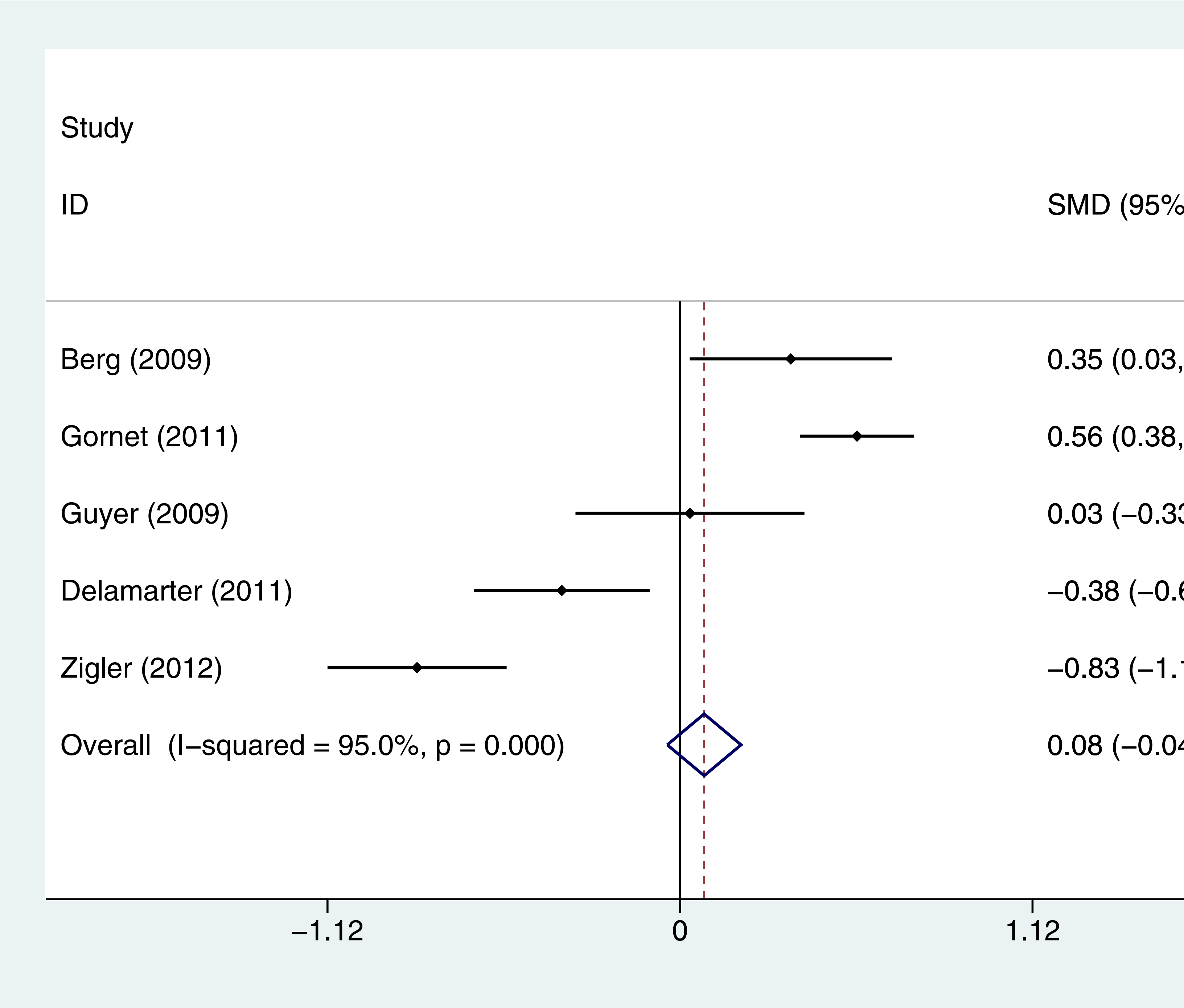
Study
ID
SMD (95%
Berg (2009)
0.35 (0.03,
Gornet (2011)
0.56 (0.38,
Guyer (2009)
0.03 (–0.33
Delamarter (2011)
–0.38 (–0.6
Zigler (2012)
–0.83 (–1.1
Overall (I–squared = 95.0%, p = 0.000)
0.08 (–0.04
–1.12
0
1.12

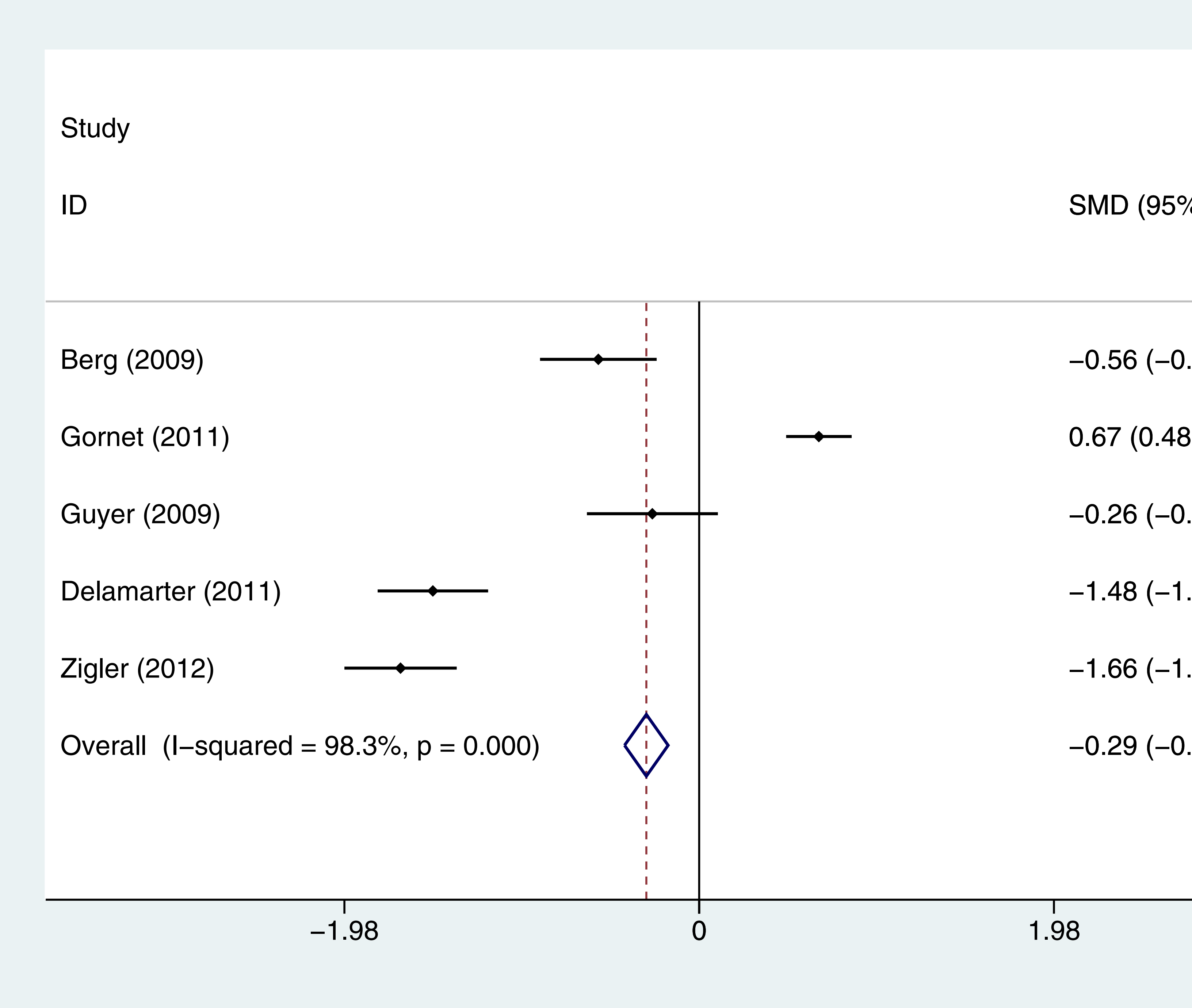

Study
ID
SMD (95%
Berg (2009)
−0.56 (−0.
Gornet (2011)
0.67 (0.48
Guyer (2009)
−0.26 (−0.
Delamarter (2011)
−1.48 (−1.
Zigler (2012)
−1.66 (−1.
Overall (I−squared = 98.3%, p = 0.000)
−0.29 (−0.
−1.98
0
1.98

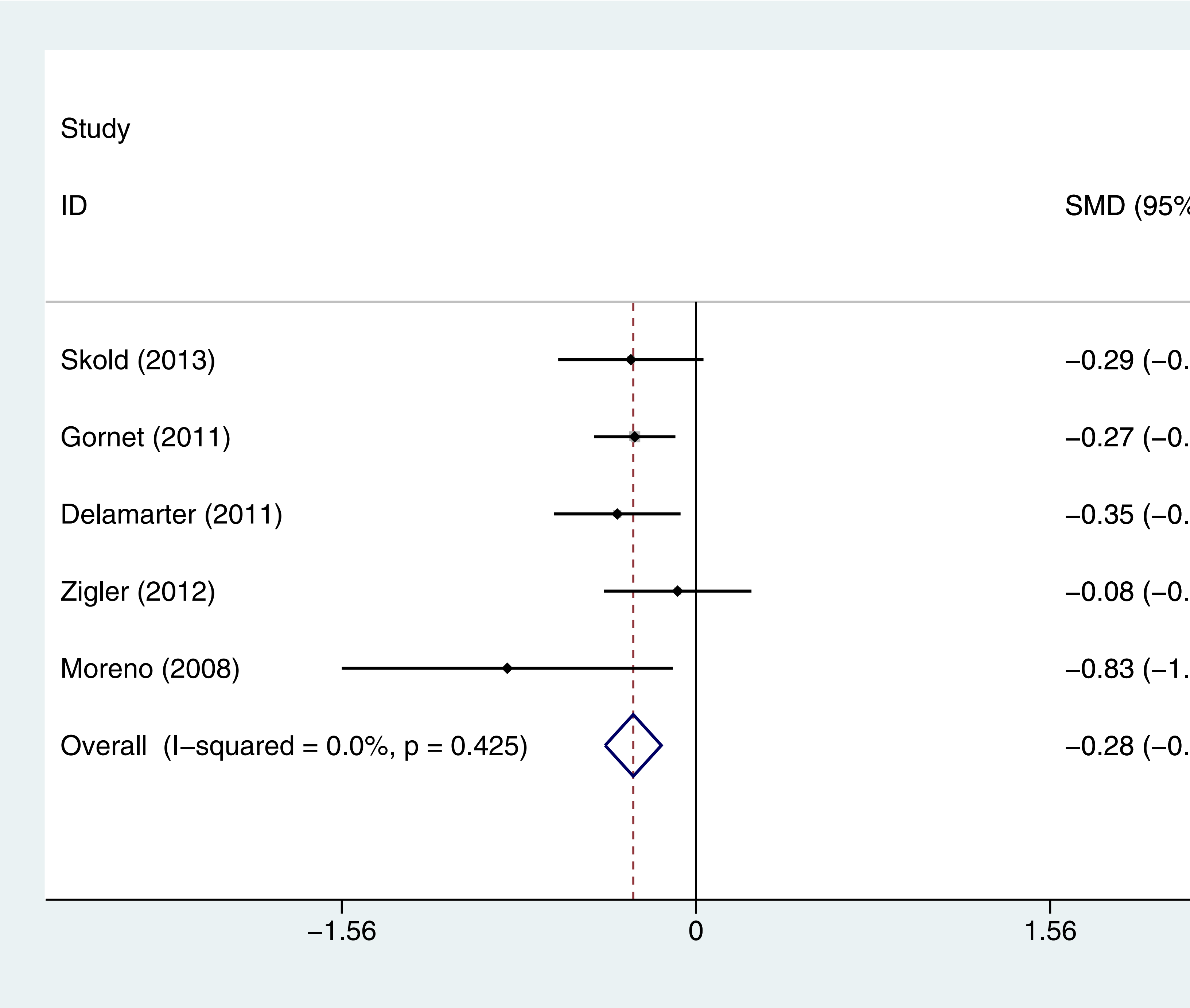

Study
ID
SMD (95%
Skold (2013)
−0.29 (−0.
Gornet (2011)
−0.27 (−0.
Delamarter (2011)
−0.35 (−0.
Zigler (2012)
−0.08 (−0.
Moreno (2008)
−0.83 (−1.
Overall (I−squared = 0.0%, p = 0.425)
−0.28 (−0.
−1.56
0
1.56
